# Identification of a Gene-Related Risk Signature in Melanoma Patients Using Bioinformatic Profiling

**DOI:** 10.1155/2020/7526204

**Published:** 2020-04-29

**Authors:** Jing Wang, Peng-Fei Kong, Hai-Yun Wang, Di Song, Wen-Qing Wu, Hang-Cheng Zhou, Hai-Yan Weng, Ming Li, Xin Kong, Bo Meng, Zong-Ke Chen, Jing-Jing Chen, Chuan-Ying Li, Jian-Yong Shao

**Affiliations:** ^1^Department of Pathology, The First Affiliated Hospital of USTC, Division of Life Sciences and Medicine, University of Science and Technology of China, Hefei, Anhui 230001, China; ^2^Department of Surgery, Fudan University Shanghai Cancer Center, Shanghai 200032, China; ^3^Department of Oncology, Shanghai Medical College, Fudan University, Shanghai 200032, China; ^4^State Key Laboratory of Oncology in South China, Collaborative Innovation Center for Cancer Medicine, Guangdong Key Laboratory of Nasopharyngeal Carcinoma Diagnosis and Therapy, Sun Yat-sen University Cancer Center, No. 651 Dongfeng East Road, Guangzhou 510060, China; ^5^Department of Molecular Diagnostics, Sun Yat-sen University Cancer Center, Guangzhou 510060, China; ^6^Department of Heart Medicine, Guangzhou Institute of Pediatrics, Guangzhou Women and Children's Medical Centre, Guangzhou Medical University, Guangzhou, Guangdong, China

## Abstract

**Introduction:**

Gene signature has been used to predict prognosis in melanoma patients. Meanwhile, the efficacy of immunotherapy was correlated with particular genes expression or mutation. In this study, we systematically explored the gene expression pattern in the melanoma-immune microenvironment and its relationship with prognosis.

**Methods:**

A cohort of 122 melanoma cases with whole-genome microarray expression data were enrolled from the Gene Expression Omnibus (GEO) database. The findings were validated using The Cancer Genome Atlas (TCGA) database. A principal component analysis (PCA), gene set enrichment analysis (GSEA), and gene oncology (GO) analysis were performed to explore the bioinformatic implications.

**Results:**

Different gene expression patterns were identified according to the clinical stage. All eligible gene sets were analyzed, and the 8 genes (GPR87, KIT, SH3GL3, PVRL1, ATP1B1, CDAN1, FAU, and TNFSF14) with the greatest prognostic impact on melanoma. A gene-related risk signature was developed to distinguish patients with a high or low risk of an unfavorable outcome, and this signature was validated using the TCGA database. Furthermore, the prognostic significance of the signature between the classified subgroups was verified as an independent prognostic predictor of melanoma. Additionally, the low-risk melanoma patients presented an enhanced immune phenotype compared to that of the high-risk gene signature patients.

**Conclusions:**

The gene pattern differences in melanoma were profiled, and a gene signature that could independently predict melanoma patients with a high risk of poor survival was established, highlighting the relationship between prognosis and the local immune response.

## 1. Introduction

To date, many advancements in melanoma have elucidated the positive and negative relationships between various clinicopathological features and prognosis. For instance, metastasis accounts for over 90% of cancer-specific mortality in melanoma [1, 2]. According to recent whole-genome mRNA expression profiling studies, melanoma can be divided into molecular subtypes, and several subtypes share clinical properties and gene expression patterns [3, 4]. Since the survival rate of melanoma patients does not significantly improve after standard treatment, the novel approach of immunotherapy is currently under intensive investigation [5, 6]. In addition, several gene patterns in melanoma have been reported to predict the strength of the antitumor response [7, 8], further highlighting the importance of precise gene signature stratification in predicting immunotherapy outcomes. However, only a few studies have systematically explored the gene expression pattern in the melanoma-immune microenvironment and its relationship with prognosis. Altogether, a better understanding of the molecular characteristics of melanoma is highly significant.

In this study, we profiled the gene expression patterns in 122 melanoma patients using whole-genome expression data from the Gene Expression Omnibus (GEO) database. Distinct degrees of phenotype enrichment were established based on the clinical stage. Using the enriched gene signature in melanoma, we found a gene-related risk signature by profiling the whole gene set, and this signature was subsequently validated using The Cancer Genome Atlas (TCGA) database. Our gene-related risk signature can independently identify melanoma patients at high risk of unfavorable clinical outcomes, and the expression intensity of immune-related genes is severely reduced in these patients, thereby indicating that survival is closely associated with the melanoma-immune microenvironment.

## 2. Materials and Methods

### 2.1. Patient Samples

In total, 581 melanoma samples from the Gene Expression Omnibus (GEO) and The Cancer Genome Atlas (TCGA) database were included in our study (Supplementary Tables 1 and 2) [9, 10]. The GEO and TCGA gene expression profiles (RNA-Seq expression) and corresponding clinical metadata were accessed from the GEO (https://www.ncbi.nlm.nih.gov/geo/) and TCGA (https://tcga-data.nci.nih.gov/tcga/dataAccess-Matrix.htm) public access databases released before May 20, 2017. The overall survival (OS) was defined from the date of diagnosis until death or the end of follow-up.

### 2.2. Standard Protocol Approval, Registration, and Patient Consent

This study was approved by the Ethics Committee and Institutional Review Board of SYSUCC. All enrolled patients signed informed consent forms.

### 2.3. Principal Components Analysis (PCA), Gene Set Enrichment Analysis (GSEA), and Gene Oncology (GO) Analysis

For each subject we used principal components analysis (PCA) to recover a low-dimensional semantic space from category model weights and classify the gene signature patterns in the patients. As previously studies reported, participants' scores in all assessments were entered into the PCA with maximum fluctuations [11]. Next, we use the degrees of freedom significance threshold (*p* < 0.05 for multiple comparisons uncorrected) to select all voxels that the model predicts significantly [12]. We then applied PCA to the category model weights of the selected voxels. A GSEA (http://www.broadinstitute.org/gsea/index.jsp) was conducted to determine whether the identified sets of genes significantly differed between the groups [13]. A GO enrichment analysis of the differentially expressed genes in the gene expression network was conducted. The DAVID database (Database for Annotation, Visualization and Integration Discovery, http://david.abcc.ncifcrf.gov/) was used to conduct a functional enrichment analysis in our study [14, 15]. Furthermore, the normalized enrichment score (NES) and false discovery rate (FDR) were applied to determine the significant differences. A *p*-value <0.05 was set as the threshold.

### 2.4. Statistical Analysis

Using the RNA-Seq database, the log 2 expression values were calculated for each probe [16]. For genes with several probes, the median was calculated for further analysis. A univariate Cox regression analysis was performed to evaluate the significance of the prognostic value of the genes in melanoma. We found 8 genes that were highly correlated with the OS (*p* < 0.01), and these genes were either associated with risk or protective based on their hazard ratio (HR). An 8-gene risk signature model was established for the prediction of survival, a univariate Cox regression analysis was conducted and a linear combination of their expression levels weighted using the regression coefficients was determined with the OS as the dependent variable [17]. Next, the melanoma patients from both the GEO and TCGA datasets were divided into high- and low-risk groups based on their median protection value. Both univariate and multivariate Cox regression analyses were performed to identify the independent prognostic factors. The primary endpoint was calculated using the Kaplan–Meier method, and the survival curves were compared using a 2-tailed log-rank test. Additionally, the differences in the clinicopathological features between the groups were evaluated using Fisher's exact test or *χ*^2^ tests. All statistical analyses were conducted using SPSS software (Version 19.0, SPSS Inc.) and GraphPad Prism (Version 5.0, GraphPad Software Inc.). A 2-sided *p*-value < 0.05 was considered statistically significant. The PCA and the generation of the heatmap and Circos diagrams were performed using R software (Version 3.4.2).

## 3. Results

### 3.1. Enhanced Gene Expression in Primary and Metastatic Melanoma

We analyzed 122 primary and metastatic melanoma cases obtained from the GEO database (GSE59455) using mRNA expression and clinical data (Supplementary Table 1). A clinical diagnosis was achieved and defined in 37 and 85 patients in primary and metastatic status, respectively. We downloaded all gene sets (hallmark and C1 to C7) from the Molecular Signatures Database (http://software.broadinstitute.org/gsea/downloads.jsp) and combined the gene sets to obtain a total gene set containing more than 20000 genes [18].

**Figure 1:**
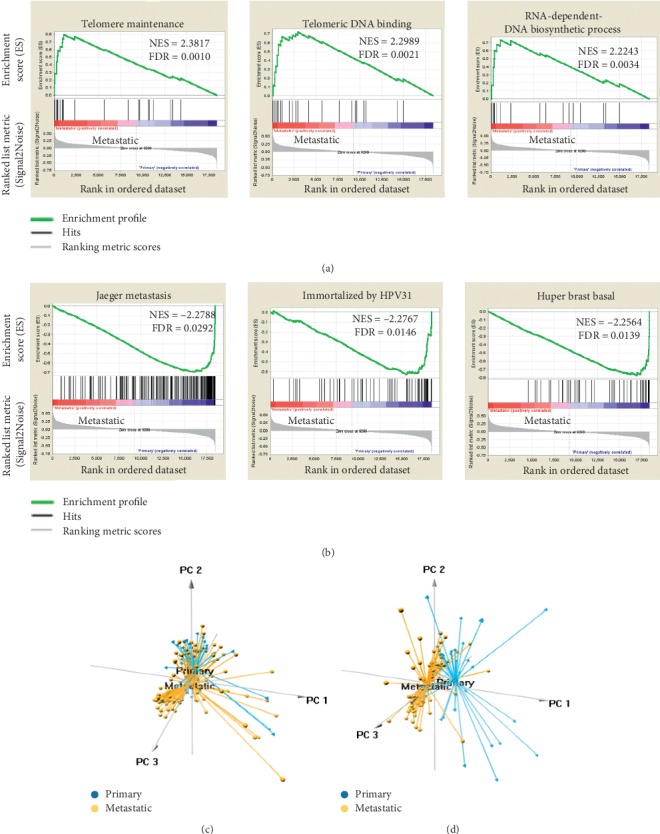
Different gene expression patterns between primary and metastatic melanoma. (a, b) Gene set enrichment analysis (GSEA) was performed to compare the gene expression between metastatic and primary tumors. FDR = false discovery rate; NES = normalized enrichment score. (c) Principal components analysis of the whole genome between primary and metastatic melanoma. (d) Principal components analysis of enriched genes between primary and metastatic melanoma.

Because the clinical and biological differences have been well established (Supplementary Table 3), we objected to further explore the different gene patterns between primary and metastatic melanoma. All gene sets were used to perform a GSEA analysis. A significantly different enrichment in the DNA binding-related, metastasis-related, and other gene sets was observed (Figures 1(a), 1(b), and Supplementary Tables 4 and 5), revealing an entirely different gene signature between the two groups. As shown in Figure 1(c), the PCA based on the whole-genome expression data showed a different intertwined pattern. Furthermore, a PCA based on the enrichment gene set data showed a relatively different distribution pattern (Figure 1(d)). Primary melanoma was distributed on the left side, while metastatic melanoma was distributed on the right side, indicating remarkably distinct gene expression patterns between the clinical stages.

### 3.2. Identification of a Local Gene Signature to Predict Prognosis in Melanoma Patients

**Figure 2:**
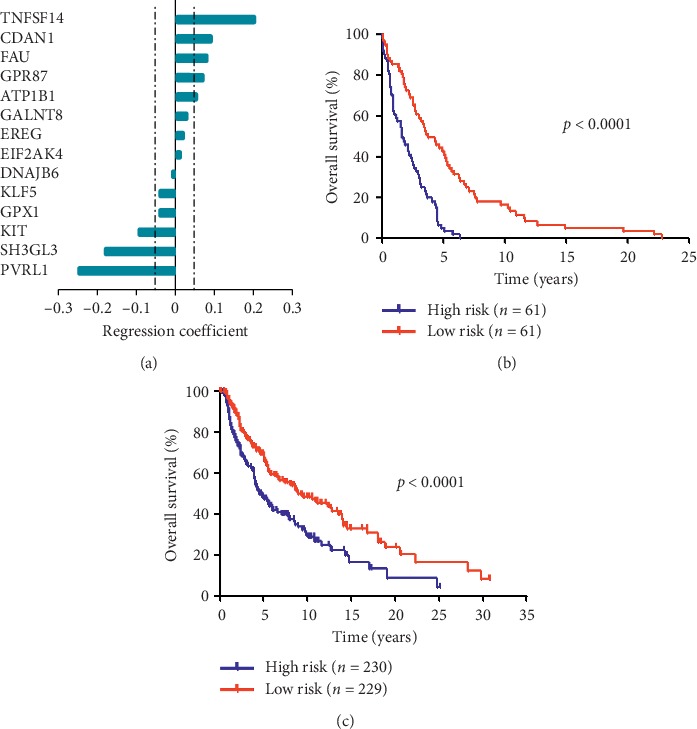
An 8-gene local gene signature in patients with melanoma. (a) The dashed lines represent an absolute regression coefficient of ±0.05. The prediction model is based on the weighted expression of eight genes and is expressed by the following equation: Protection score = (0.202 × TNFSF14) + (0.091 × CDAN1) + (0.081 × FAU) + (0.071 × GPR87) + (0.052 × ATP1B1) + (−0.094 × KIT) + (−0.179 × SH3GL3) + (−0.250 × PVRL1). (b) Survival curves of overall survival in high- and low-risk groups classified by the local gene signature in the GEO database. (c) Survival curves of overall survival in high- and low-risk groups classified by the local gene signature in the TCGA database.

Considering the enrichment gene expression in primary and metastatic melanoma, we attempted to establish a local gene signature as a predictor of prognosis. Subsequently, we performed a univariate Cox regression analysis to explore the prognostic value of these enriched genes. In our study, fourteen genes (EREG, GALNT8, GPR87, KIT, KLF5, SH3GL3, PVRL1, ATP1B1, CDAN1, DNAJB6, EIF2AK4, FAU, GPX1, and TNFSF14) were observed to predict survival in melanoma (Supplementary Table 6, *p* < 0.01). Then, the risk score method was used to establish a risk signature for melanoma patients based on the gene expression levels [17]. As presented in Figure 2(a), we ranked the genes based on their predictive power (regression coefficients). We excluded several genes with relatively low predictive power (−0.05 < regression coefficient < 0.05). Finally, eight genes (GPR87, KIT, SH3GL3, PVRL1, ATP1B1, CDAN1, FAU, and TNFSF14) were identified to be closely associated with the OS in melanoma. In addition, all identified genes were of the following 2 types: risky or protective. An HR > 1 was defined as risky (GPR87, KIT, SH3GL3, and PVRL1), and an HR < 1 was defined as protective (ATP1B1, CDAN1, FAU, and TNFSF14).

The prediction model is based on the weighted expression of eight genes and is expressed by the following equation: protection value score = (0.202 × TNFSF14) + (0.091 × CDAN1) + (0.081 × FAU) + (0.071 × GPR87) + (0.052 × ATP1B1) + (−0.094 × KIT) + (−0.179 × SH3GL3) + (−0.250 × PVRL1). All cases were divided into high-risk (*n* = 61) and low-risk (*n* = 61) subgroups based on the median protection value as the cut-off. Compared with the low-risk patients, the high-risk patients are associated with a shorter OS (High-risk vs. low-risk: median OS, 1.66 vs. 3.82 years; HR = 3.14, 95% confidence interval [CI] 2.07 to 4.78; *p* < 0.0001; Figure 2(b)). To validate the prognosis prediction of the 8-gene-based gene signature, we calculated the protection value of each patient in the TCGA database using the same formula. Similarly, the patients were classified into high- and low-risk groups using the same method. Expectedly, the OS in the high-risk group was shorter than that in the low-risk group (High-risk vs. low-risk: median OS, 1.66 vs. 3.82 years; HR = 1.75, 95% CI 1.33 to 2.30; *p* < 0.0001; Figure 2(c)).

### 3.3. Correlations between the Local Gene Signature and Prognostic Features in Melanoma

The baseline characteristics of the GEO and TCGA cohort were compared based on the local gene signature, and the comparisons are shown in Supplementary Tables 7 and 8. Overall, in the TCGA cohort, the age at diagnosis (High-risk vs. low-risk: mean age, 55.7 vs. 60.5 years, *p*=0.002), advanced AJCC (American Joint Committee on Cancer) stage (High-risk vs. low-risk: rate, 55.3% vs. 35.7%, *p* < 0.001), and NRAS mutation rate (High-risk vs. low-risk: rate, 32.2% vs. 23.1%, *p*=0.037) significantly differed between the high- and low-risk groups (Supplementary Table 7). The GEO cohort exhibited a similar distribution (Supplementary Table 8). Next, we performed univariate and multivariate Cox regression analyses using the TGGA database and revealed that the local gene-related risk signature was independently correlated with OS (Table 1). Furthermore, the local gene-related risk signature was validated as an independent factor using the GEO database (Supplementary Table 9), confirming that this signature independently predicts prognosis with strong power.

**Figure 3:**
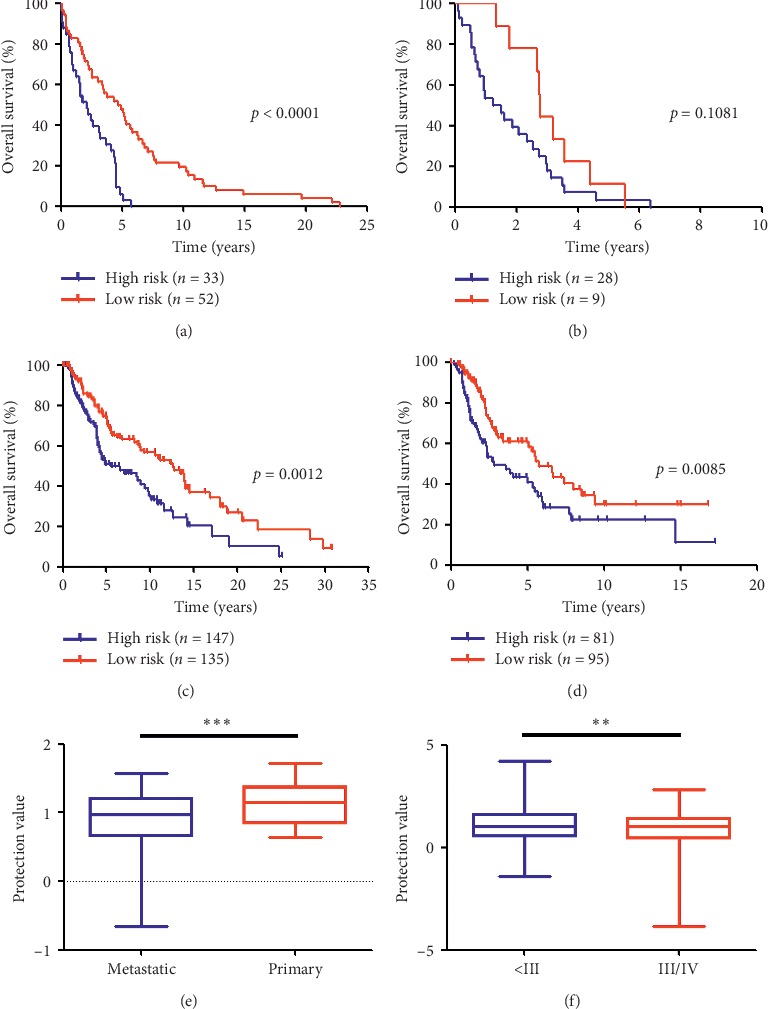
Associations between the local gene signature and clinicopathological features in melanoma. (a) Survival curves of overall survival in high- and low-risk groups classified by the local gene signature in metastatic melanoma patients (GEO database). (b) Survival curves of overall survival in high- and low-risk groups classified by the local gene signature in primary melanoma patients (GEO database). (c) Survival curves of overall survival in high- and low-risk groups classified by the local gene signature in AJCC stage I/II melanoma patients (TCGA database). (d) Survival curves of overall survival in high- and low-risk groups classified by the local gene signature in stage III/IV melanoma patients (TCGA database). (e) Associations between the protection value and the clinicopathological features (Primary vs. Metastatic). (f) Associations between the protection value and the clinicopathological features (Stage I/II vs. Stage III/IV).

In addition, as shown by the Kaplan–Meier OS curves (Figure 3(a)), the OS during early-stage melanoma significantly differed between the high- and low-risk groups of patients in the GEO datasets (High-risk vs. low-risk: median OS, 2.15 vs. 4.84 years; HR = 3.50, 95% CI 2.00 to 6.13; *p* < 0.0001). Additionally, although not statistically significant, a divergence appeared to emerge in the OS curves prior to 10 years of follow-up in patients at the advanced-stage (High-risk vs. low-risk: median OS, 1.38 vs. 2.79 years; HR = 1.75, 95% CI 0.88 to 3.48; *p*=0.1081; Figure 3(b)). Subsequently, we validated our novel findings using the TCGA database. During both the early (High-risk vs. low-risk: median OS, 5.56 vs. 12.61 years; HR = 1.82, 95% CI 1.27 to 2.60; *p*=0.0012; Figure 3(c)) and advanced (High-risk vs. low-risk: median OS, 2.86 vs. 5.76 years; HR = 1.78, 95% CI 1.16 to 2.75; *p*=0.0085; Figure 3(d)) stages, the gene signature had prognostic significance. Furthermore, the signature protection value differed between patients stratified by clinical and AJCC stages (Figures 3(e) and 3(f)).

### 3.4. Application of the Local Gene Signature in Stratified Melanoma Cohorts

**Figure 4:**
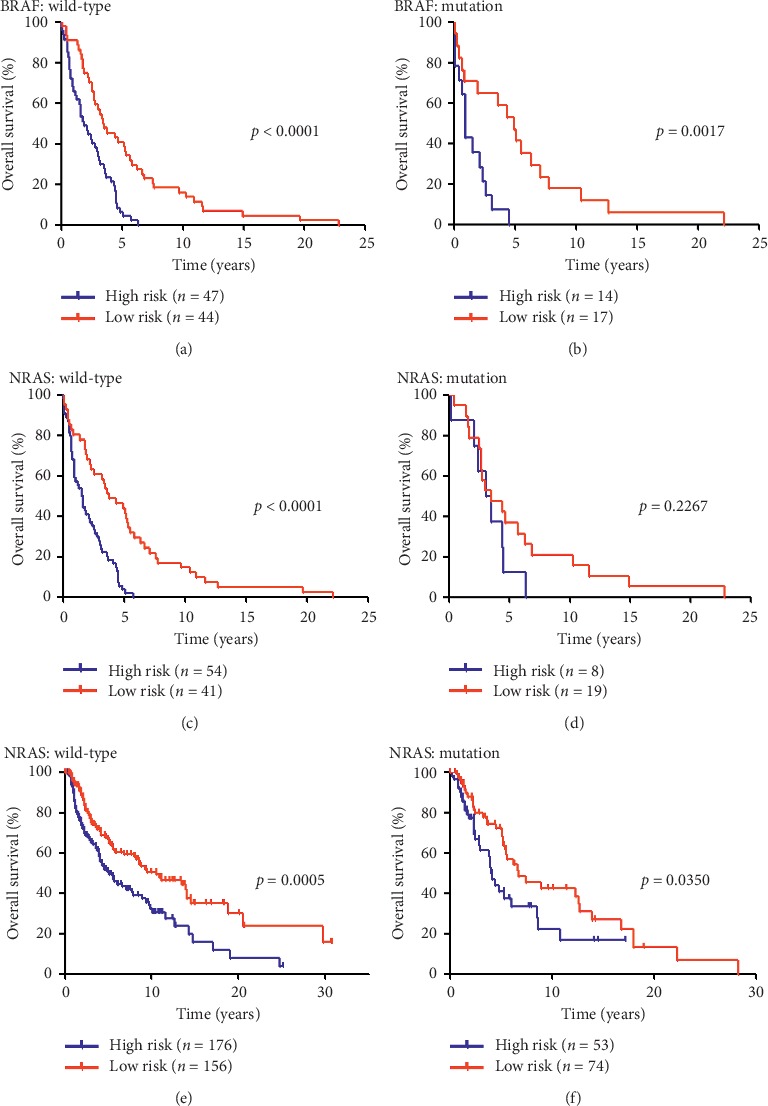
Application of the local gene signature in stratified melanoma cohorts. (a) Survival curves of overall survival in high- and low-risk groups classified by the local gene signature in BRAF wild-type melanoma patients (GEO database); (b) survival curves of overall survival in high- and low-risk groups classified by the local gene signature in BRAF mutation melanoma patients (GEO database); (c) survival curves of overall survival in high- and low-risk groups classified by the local gene signature in NRAS wild-type melanoma patients (GEO database); (d) survival curves of overall survival in high- and low-risk groups classified by the local gene signature in NRAS mutation melanoma patients (GEO database); (e) survival curves of overall survival in high- and low-risk groups classified by the local gene signature in NRAS wild-type melanoma patients (TCGA database); (f) survival curves of overall survival in high- and low-risk groups classified by the local gene signature in NRAS mutation melanoma patients (TCGA database).

In this study, we evaluated the prognostic value of the local gene signature in stratified cohorts. The melanoma patients were first classified according to the status of BRAF and NRAS mutation. In the GEO cohort, the high-risk patients had a significantly shorter OS than the low-risk patients (Figures 4(a)–4(c)), except for the NRAS mutation cohort (High-risk vs. low-risk: median OS, 3.34 vs. 3.57 years; HR = 1.82, 95% CI 0.69 to 4.81; *p*=0.2267; Figure 4(d)). Subsequently, we validated these new findings using the TCGA database. Similarly, patients with BRAF and NRAS status were selected to validate the local gene expression patterns, and the OS in the high-risk group was shorter than that in the low-risk group in all cohorts (Figures 4(e), 4(f) and Supplementary Figures 5(a) and 5(b)). Taken together, the gene risk signature-based classification could accurately identify patients with poor prognosis regardless of the BRAF and NRAS status.

### 3.5. Low-Risk Melanoma Patients Exhibited an Enhanced Local Immune Phenotype

**Figure 5:**
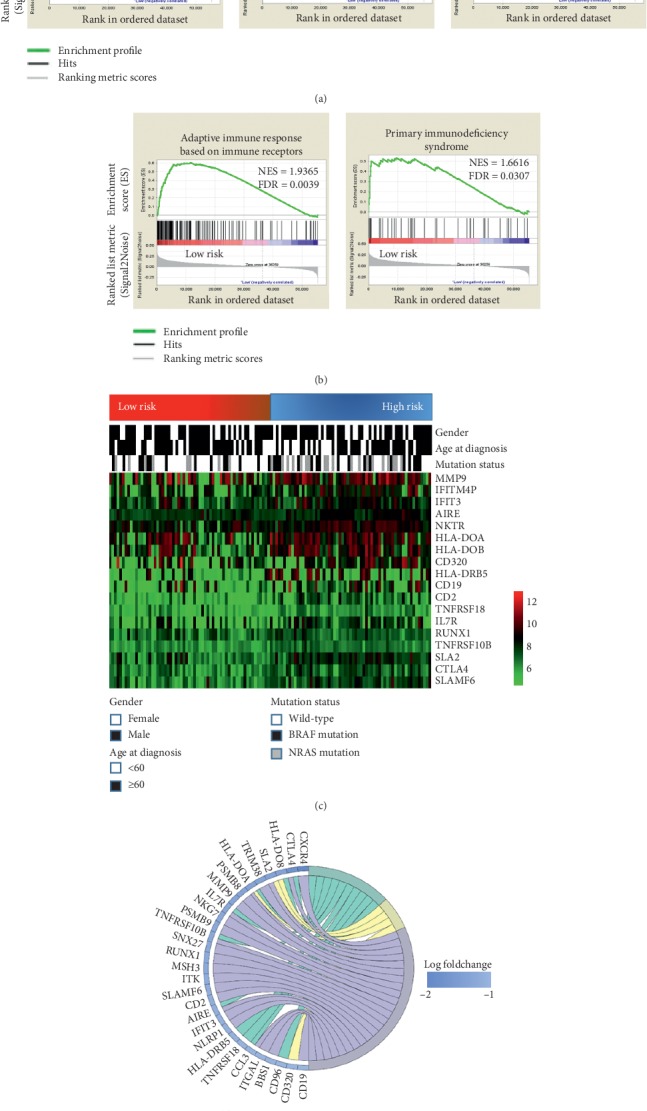
Immune signature differences between high- and low-risk groups in melanoma. (a, b) Significant enrichment of the immune-related phenotype in low-risk patients compared to high-risk patients. TGGA = The Cancer Genome Atlas; FDR = false discovery rate; NES = normalized enrichment score; (c) associations between the protection value and the clinicopathological features and immune-related genes; (d) Circos diagram of the gene expression involved in immune response pathways.

Considering the distinct prognosis based on gene signature, we explored the phenotypical differences between the risk groups using genome expression data. Melanoma is known as the most common immune-related malignancy, and melanoma patients were the first to benefit from immunotherapy. Hence, the five most common immune-related gene sets (adaptive immune response M13847, activation of immune response M19789, activation of immune innate response M15340, adaptive immune response based on immune receptors M11342, and primary immunodeficiency syndrome M7603) were extracted from the Molecular Signatures Database, and an immune-related gene set was created. Interestingly, compared with the high-risk group, the GSEA revealed a highly significant enrichment of immune-related phenotypes in the low-risk group (Figures 5(a) and 5(b)), indicating that patients with the low-risk gene signature had an intense local immune response microenvironment. Next, the patients in the GEO database were divided into high- and low-risk groups according to their protection values. As presented in Figure 5(c), the genes forming the gene risk signature exhibited distinct expression patterns that corresponded to the protection value. The low-risk patients exhibited high expression levels of T cell activation-related genes (TNFSF14, AIRE, CD2, and CD19), NK cell activation-related genes (SLAMF6 and NKTR), and autoimmune-related genes (Figure 5(c)). Additionally, the low-risk patients had higher expression levels of a crucial negative regulator of the immune system (CTLA4) and a protective gene (TNFRSF10B). However, the signature value did not differ between the cases stratified by age at diagnosis, gender, and molecular subtype. Furthermore, the Circos diagram of the GO analysis illustrates the identical tendency of the immune-related genes between the two groups (Figure 5(d)).

## 4. Discussion

In this study, we firstly identified a gene signature that was significantly associated with OS in patients with melanoma using gene expression data from the GEO and TCGA databases. Furthermore, different immune gene patterns were observed in high- and low-risk patients. In the patients with the low-risk gene signature, the innate and adaptive immune systems are capable of coordinating a robust immune response, indicating a need for a distinct immunotherapy strategy according to the gene expression pattern.

The identification of molecular subtypes in other malignancies provided the impetus to utilize transcriptome profiling to explore the gene expression patterns in melanoma. Previous studies have used mRNA expression profiling to distinguish among the subtypes of lymphoma with a high degree of accuracy [19, 20]. Importantly, parallel studies in melanoma also revealed that patients could be grouped into “molecular subtypes” with very different biological properties that clinically behave as different disease entities [3, 4, 7, 21, 22]. In the present study, a large sample of melanoma cases from the GEO database, including 37 primary and 85 metastatic melanoma cases, was used as a discovery set. In the preliminary analysis, markedly distinct local gene phenotypes were observed based on the clinical stage (primary vs. metastatic), particularly in telomere maintenance, telomeric DNA binding, biosynthetic process, and metastasis (Figures 1(a), 1(b) and Supplementary Figures 1–4). Consistent results have been reported in previous studies, in which telomere maintenance, cancer metabolism, and DNA repair were highly associated with malignancy progression, and poor clinical outcomes were predominant in melanoma with high malignancy [23–25]. However, differences across the entire gene set have not been identified in melanoma patients at different stages. Accordingly, we are the first to demonstrate that the overall gene expression pattern in melanoma patients is positively distinguished by the malignant grade (Figures 1(c) and 1(d)).

To the best of our knowledge, establishing precise signatures to determine the status of patients is refreshing because these signatures are powerful prognostic predictors and, if correctly applied, can enable patient stratification to achieve better immunotherapeutic outcomes. Numerous studies have investigated both single prognostic biomarkers and local immune parameters in patients with melanoma [26, 27]. However, the prognostic value of systemic gene signatures remains unclear. In our study, we identified two gene expression patterns and generated an 8-gene-based (GPR87, KIT, SH3GL3, PVRL1, ATP1B1, CDAN1, FAU, and TNFSF14) gene signature that could recognize melanoma patients with a high risk of unfavorable clinical outcomes. Next, we tested the signature using the GEO database (for discovery) and validated the signature using the TCGA database (Figures 2(a)–2(c)). Our signature consists of diverse genes comprising protective (ATP1B1, CDAN1, FAU, and TNFSF14)) and risky (GPR87, KIT, SH3GL3, and PVRL1), which could be considered gene-related protective and risk patterns in melanoma. Altogether, our findings may prompt a novel treatment strategy to improve prognosis by shaping the gene signature. According to previous studies, the genes forming our signature could be considered promising therapeutic targets due to their nature and prognostic impact. KIT, which is a famous oncogene, is often mutated in advanced melanoma and contributes to malignancy progression and an unfavorable prognosis [28, 29], while highly expressed TNFSF14 in human melanoma cells and microvesicles may contribute to the mediation of T cell responses to cancer cells [30]. Notably, although the available genomic and associated clinical data have been verified, several genes constituting our signature have not been studied in melanoma. However, these genes appear to exert oncogenic or tumor suppressive functions in other tumors. For instance, GPR87 plays a critical oncogenic role in pancreatic cancer progression, and SH3GL3 is a novel invasion-associated candidate gene that likely contributes to the invasive genotype of malignant gliomas [31, 32]. Furthermore, our gene risk signature remained an independent prognostic predictor after adjusting for the clinicopathological and molecular features (Table 1).

To better understand the gene signature influencing patient survival, we conducted a subgroup analysis and mainly focused on tumor stage (clinical or AJCC stage) and molecular characteristics (BRAF and NRAS status). In the GEO database, more than 80 metastatic melanomas were analyzed, and this sample size was sufficient to display the power of the gene signature in predicting the outcome even after adjusting for the clinical stage (Figures 3(a) and 3(b)). Furthermore, the clinical implications according to molecular grading and staging were immediately validated using the TCGA dataset (Figures 3(c) and 3(d)). Moreover, as shown in the protection value pattern presented in Figures 3(e) and 3(f), the advanced-stage melanomas underwent a malignant course in our gene expression model. In general, the BRAF and NRAS status defined the nature of the proliferative apparatus, which has been well established as a major molecular biomarker of melanoma [33, 34]. The gene-related risk feature might have contributed to the poor prognosis in the patients regardless of BRAF status in both the discovery and the validation databases (Figures 4(a), 4(b) and Supplementary Figure 5). Similarly, despite the uncertain results using the GEO dataset (Figures 4(c) and 4(d)), a Kaplan–Meier analysis of TCGA patients suggested that patients with the high-risk gene signature had a worse OS than patients with the low-risk signature in both the NRAS wild-type and mutant subgroups (Figures 4(e) and 4(f)). Considering that BRAF and NRAS mutant melanomas are prone to an ominous prognostic outcome [35, 36], the similar gene expression signature pattern of the wild-type and mutant melanomas indicates that our model-based classification could accurately identify patients with unfavorable prognoses regardless of the BRAF and NRAS status. However, the exact mechanism remains unknown and should be further examined.

Data from previous studies investigating the response of melanoma to immune checkpoint inhibitors have illustrated the need to develop a strategy to consider stratification based on the gene signature. Due to its formation of related genes, the signature was highly associated with the overall intensity of the local immune response. The designated low-risk patients exhibited an enhanced local immune phenotype compared to the low-risk patients (Figures 5(a) and 5(b)). Interestingly, the local immune signature pattern was compatible with prognosis determination in patients at a low- or high-risk, clearly suggesting that high-risk patients share similar decreased immune abilities in determining prognosis, even with the different intensity of T cell activation-related genes (TNFSF14, AIRE, CD2, and CD19), NK cell activation-related genes (SLAMF6, and NKTR), and autoimmune-related genes (HLA-DOB, HLA-DOA, IL7R, and TNFRSF18) (Figures 5(c) and 5(d)). However, this result is consistent with those of previous reports showing that the immune response against tumors increased the survival time of patients with advanced-stage tumors, such as melanoma, lung cancer, and hepatocellular carcinomas [37–39]. In addition, low-risk patients have higher expression levels of a crucial negative regulator of the immune system (CTLA4) and a protective gene (TNFRSF10B). According to preliminary reports, melanoma patients can benefit from anti-CTLA treatment [40, 41], highlighting the potential of our signature to identify melanoma patients in which the use of immune checkpoint inhibitors is effective.

Additionally, several limitations of this study must be addressed. First, our study is limited since it is retrospective and should be validated by prospective studies. Second, to achieve better clinical application, the validity of our signature in predicting responses to immunotherapy and relationship with hyperprogressive disease (HPD) should be tested; as previously reported, local immune factors are potentially involved in the formation of this signature [42]. Finally, functional and mechanistic studies should be performed to investigate the 8 genes alone or in combination to support the clinical application of our signature.

## 5. Conclusions

We identified an 8-gene signature (i.e., GPR87, KIT, SH3GL3, PVRL1, ATP1B1, CDAN1, FAU, and TNFSF14) with independent prognostic value on melanoma. Additionally, the gene expression pattern correlated with melanoma-immune microenvironment and immune-related therapy.

## Figures and Tables

**Table 1:** Multivariate Cox regression analysis of clinicopathological factors and overall survival using the TCGA database.

	Univariate Cox regression		Multivariate Cox regression	
Variable	HR	*p* value	HR	*p* value
*Age*				
Increasing years	1.748	0.001	1.517	0.018
*Sex*				
Female vs. male	0.784	0.979		
*BRAF status*				
Mutation vs. wild-type	1.174	0.035	1.142	0.089
*NRAS status*				
Mutation vs. wild-type	0.957	0.580		
*Clinical stage*				
III + IV vs. <III	1.023	0.001	1.023	0.001
*Local gene signature*				
High-risk vs. low-risk	1.318	<0.001	1.274	0.001

TCGA: The Cancer Genome Atlas. HR: hazard ratio.

## Data Availability

All the data supporting the conclusions of this article are included in the article and its supplementary information files.

## Abbreviations

GEO: Gene Expression Omnibus
TCGA: The Cancer Genome Atlas
PCA: Principal components analysis
GSEA: Gene set enrichment analysis, GO gene oncology analysis
NES: The normalized enrichment score
FDR: False discovery rate.

## Supplementary Materials

Supplementary Materials Supplementary Table 1: the characteristics of patients from the GEO database. Supplementary Table 2: the characteristics of patients from the TCGA database. Supplementary Table 3: comparison of clinicopathologic characteristics between primary and metastatic melanoma in GEO database. Supplementary Table 4: biological processes enriched in the metastatic group. Supplementary Table 5: biological processes enriched in the primary group. Supplementary Table 6: fourteen genes with prognostic value in CEO melanoma patients. Supplementary Table 7: comparison of clinicopathologic characteristics between high and low-risk melanoma in the TCGA database. Supplementary Table 8: comparison of clinicopathologic characteristics between high and low-risk melanoma in the GEO database. Supplementary Table 9: multivariate Cox regression analysis of clinicopathologic factors for overall survival in the GEO database. Supplementary Figure 1: gene set enrichment analysis (GSEA) for comparing genotype between metastatic and primary. FDR = false discovery rate; NES = normalized enrichment score. Supplementary Figure 2: gene set enrichment analysis (GSEA) for comparing genotype between metastatic and primary. FDR = false discovery rate; NES = normalized enrichment score Supplementary Figure 3: gene set enrichment analysis (GSEA) for comparing genotype between metastatic and primary. FDR = false discovery rate; NES = normalized enrichment score. Supplementary Figure 4: gene set enrichment analysis (GSEA) for comparing genotype between metastatic and primary. FDR = false discovery rate; NES = normalized enrichment score. Supplementary Figure 5: (a) survival curves of overall survival for high and low-risk groups classified by the local gene signature in BRAF wild-type melanoma patients (TCGA database); (b) survival curves of overall survival for high and low-risk groups classified by the local gene signature in BRAF mutation melanoma patients (TCGA database).
