# Supplementary material for: Identification of a Gene-Related Risk Signature in Melanoma Patients Using Bioinformatic Profiling
Source: J Oncol. 2020 Apr 29;2020:7526204. doi: 10.1155/2020/7526204 (PMC7206882; doi:10.1155/2020/7526204)
Supplement: Supplementary Materials — Supplementary Table 1: the characteristics of patients from the GEO database. Supplementary Table 2: the characteristics of patients from the TCGA database. Supplementary Table 3: comparison of clinicopathologic characteristics between primary and metastatic melanoma in GEO database. Supplementary Table 4: biological processes enriched in the metastatic group. Supplementary Table 5: biological processes enriched in the primary group. Supplementary Table 6: fourteen genes with prognostic value in CEO melanoma patients. Supplementary Table 7: comparison of clinicopathologic characteristics between high and low-risk melanoma in the TCGA database. Supplementary Table 8: comparison of clinicopathologic characteristics between high and low-risk melanoma in the GEO database. Supplementary Table 9: multivariate Cox regression analysis of clinicopathologic factors for overall survival in the GEO database. Supplementary Figure 1: gene set enrichment analysis (GSEA) for comparing genotype between metastatic and primary. FDR = false discovery rate; NES = normalized enrichment score. Supplementary Figure 2: gene set enrichment analysis (GSEA) for comparing genotype between metastatic and primary. FDR = false discovery rate; NES = normalized enrichment score Supplementary Figure 3: gene set enrichment analysis (GSEA) for comparing genotype between metastatic and primary. FDR = false discovery rate; NES = normalized enrichment score. Supplementary Figure 4: gene set enrichment analysis (GSEA) for comparing genotype between metastatic and primary. FDR = false discovery rate; NES = normalized enrichment score. Supplementary Figure 5: (a) survival curves of overall survival for high and low-risk groups classified by the local gene signature in BRAF wild-type melanoma patients (TCGA database); (b) survival curves of overall survival for high and low-risk groups classified by the local gene signature in BRAF mutation melanoma patients (TCGA database). [file 7526204.f1.pdf]

Supplementary Table 1. The characteristics of patients from the GEO database.

Variable		No. of Patients (n=122)	%
Age at diagnosis, years			
	Mean	66.4	
	SD	15.5	
Sex			
	Male	84	68.9
	Female	38	31.1
Stage			
	Primary	37	30.3
	Metastatic	85	69.7
BRAF status			
	Mutation	31	25.4
	WT	91	74.6
NRAS status			
	Mutation	27	22.1
	WT	95	77.9
Overall survival, years			
	Mean	3.8	
	SD	4.1	

Abbreviations: GEO, Gene Expression Omnibus. HR, hazard ratio. SD, Standard Deviation. WT, wild-type.

Supplementary Table 2. The characteristics of patients from the TCGA database.

**Variable**		**No. of Patients (n=459)**	**%**
Age at diagnosis, years			
	Mean	58.0	
	SD	15.9	
Sex			
	Male	284	61.9
	Female	175	38.1
AJCC stage			
	< III	249	54.2
	III	185	40.3
	IV	25	5.4
BRAF status			
	Mutation	199	43.4
	WT	260	56.6
NRAS status			
	Mutation	127	27.7
	WT	332	72.3
Overall survival, years			
	Mean	5.0	
	SD	5.3	

Abbreviations: TCGA, The Cancer Genome Atlas. SD, Standard Deviation. AJCC, American Joint Committee on Cancer, 7th Edition. WT, wild-type.

Supplementary Table 3. Comparison of clinicopathologic characteristics between primary and metastatic melanoma in GEO database.

Variable		Primary		Metastatic	
		No. of Patients (n=37)	%	No. of Patients (n=85)	%
Age at diagnosis, years					
	Mean	75.1		62.8	
	SD	14.3		14.8	
Sex					
	Male	26	70.3	58	68.2
	Female	11	29.7	27	31.8
BRAF Mutation					
	Mutation	8	21.6	23	27.1
	WT	29	78.4	62	72.9
NRAS Mutation					
	Mutation	7	18.9	20	23.5
	WT	30	81.1	65	76.5
Overall survival, years					
	Mean	2.1		4.5	
	SD	1.5		4.7	

Abbreviations: GEO, Gene Expression Omnibus. SD, Standard Deviation. WT, wild-type.

Supplementary Table 4. Biological processes enriched in metastatic group.

**Biological process**	**Size**	**NES**	**FDR**
Telomere maintenance	21	2.3817	0.0010
Telomeric DNA binding	26	2.2989	0.0021
RNA-dependent-DNA biosynthetic process	17	2.2243	0.0034
Regulation of endopeptidase activity	87	2.1546	0.0012
Regulation of DNA replication	50	2.1081	0.0198
Ribosomal large subunit assembly	19	2.1053	0.0170
Ribosomal subunit	150	2.0568	0.0344
Metabolic process	132	2.0508	0.0330
Cytosolic ribosome	100	2.0253	0.0385
ATPase activator activity	17	2.0213	0.0376

Abbreviations: NES, normalized enrichment score. FDR , false discovery rate .

Supplementary Table 5. Biological processes enriched in primary group.

**Biological process**	**Size**	**NES**	**FDR**
Jaeger metastasis	241	-2.2788	0.0292
Immortalized by HPV31	64	-2.2767	0.0146
Huper brast basal	53	-2.2564	0.0139
Module 438	64	-2.3025	0.0048
Module 153	34	-2.2607	0.0055
Module 297	76	-2.2392	0.0057
Module 357	76	-2.2119	0.0062
Module 154	71	-2.2010	0.0054
Module 298	25	-2.1626	0.0103
Cell differentiation	125	-2.2650	0.0092
Desmosome	24	-2.2592	0.0050
Keratinocyte differentiation	89	-2.2454	0.0048
Skin development	199	-2.2181	0.0077
Epidermis development	233	-2.1883	0.0117
Peptide cross linking	51	-2.1565	0.0179
Cell adherence unction	51	-2.1209	0.0254
KRAS lung	133	-1.9376	0.0184

Abbreviations: NES, normalized enrichment score. FDR, false discovery rate .

Supplementary Table 6. Fourteen genes with prognostic value in CEO melanoma patients..

Gene	HR	95% CI	P value
EREG	1.391	1.096 to 1.766	0.007
GALNT8	1.408	1.098 to 1.804	0.007
GPR87	1.314	1.086 to 1.591	0.005
KIT	1.268	1.068 to 1.505	0.007
KLF5	1.630	1.117 to 2.257	0.003
SH3GL3	1.493	1.233 to 1.809	<0.001
PVRL1	1.612	1.192 to 2.178	0.002
ATP1B1	0.749	0.607 to 0.923	0.007
CDAN1	0.544	0.383 to 0.772	0.001
DNAJB6	0.748	0.612 to 0.913	0.004
EIF2AK4	0.772	0.640 to 0.930	0.006
FAU	0.774	0.659 to 0.909	0.002
GPX1	0.765	0.630 to 0.928	0.006
TNFSF14	0.570	0.433 to 0.751	<0.001

Abbreviations: GEO, Gene Expression Omnibus. HR, hazard ratio.

Supplementary Table 7. Comparison of clinicopathologic characteristics between high and low risk melanoma in TCGA database.

Variable	High risk		Low risk		p value
	No. of Patients (n=230)	%	No. of Patients (n=229)	%	
Age at diagnosis, years					0.002
Mean	55.7		60.5		
SD	16.3		15.2		
Sex					0.565
Male	139	60.4	145	63.3	
Female	91	39.6	84	36.7	
AJCC stage					<0.001
< III	105	44.7	144	64.3	
III+IV	130	55.3	80	35.7	
BRAF Mutation					0.572
Mutation	103	44.8	96	41.9	
WT	127	55.2	133	58.1	
NRAS Mutation					0.037
Mutation	74	32.2	53	23.1	
WT	156	67.8	176	76.9	

Abbreviations: TCGA, The Cancer Genome Atlas. SD, Standard Deviation. AJCC, American Joint Committee on Cancer, 7th Edition. WT, wild-type.

Supplementary Table 8. Comparison of clinicopathologic characteristics between high and low risk melanoma in GEO database.

**Variable**	**High risk**		**Low risk**		**p value**
	**No. of Patients (n=61)**	**%**	**No. of Patients (n=61)**	**%**	
Age at diagnosis, years					0.183
Mean	68.7		64.7		
SD	15.1		16.0		
Sex					1.000
Male	42	68.9	41	67.2	
Female	19	31.1	20	32.8	
Clinical stage					<0.001
Primary	28	45.9	52	85.2	
Metastatic	33	54.1	9	14.8	
BRAF Mutation					0.678
Mutation	14	21.6	17	27.9	
WT	47	78.4	44	72.1	
NRAS Mutation					0.008
Mutation	7	11.5	20	32.8	
WT	54	88.5	41	67.2	

Abbreviations: GEO, Gene Expression Omnibus. SD, Standard Deviation. WT, wild-type.

Supplementary Table 9. Multivariate Cox regression analysis of clinicopathologic factors for overall survival in GEO database.

	Univariate cox		Multivariate cox	
**Variable**	**HR**	**p Value**	**HR**	**p Value**
Age				
Increasing years	1.600	0.014	1.193	0.076
Sex				
Female vs male	0.920	0.392		
BRAF status				
Mutation vs wild-type	0.981	0.860		
NRAS status				
Mutation vs wild-type	0.803	0.048	0.822	0.087
Clinical stage				
Metastatic vs primary	2.183	<0.001	1.516	0.031
Local gene signature				
High risk vs low risk	1.721	<0.001	1.570	<0.001

Abbreviations: GEO, Gene Expression Omnibus. HR, hazard ratio.

Supplementary Figure 1

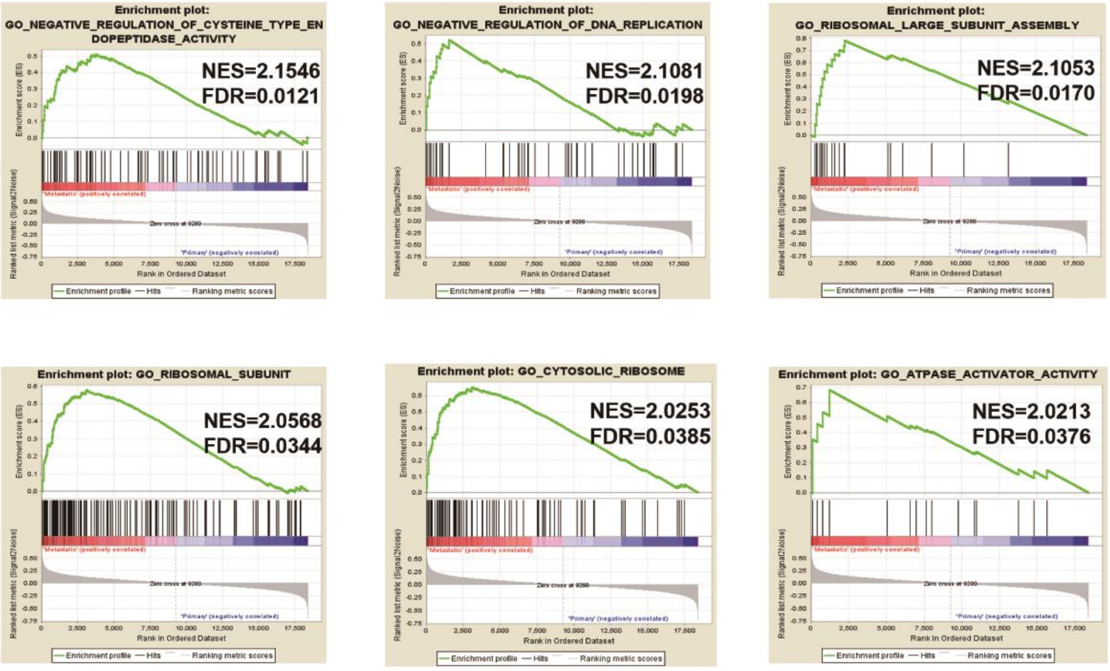

Gene set enrichment analysis (GSEA) for comparing genotype between metastatic and primary. FDR=false discovery rate; NES=normalized enrichment score.

Supplementary Figure 2

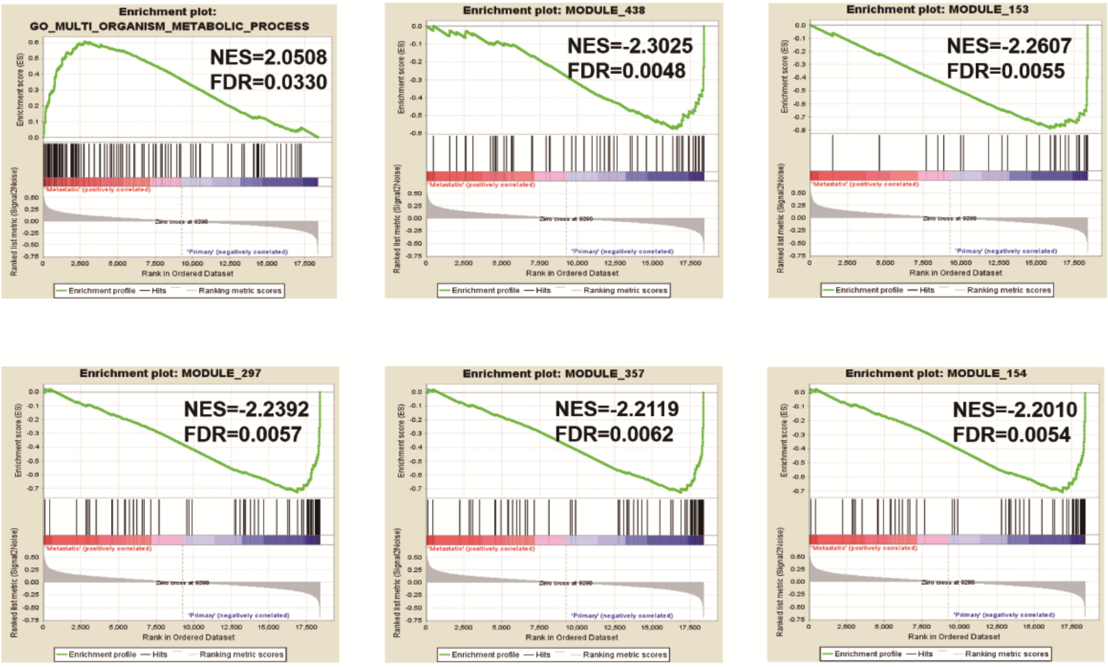

Gene set enrichment analysis (GSEA) for comparing genotype between metastatic and primary. FDR=false discovery rate; NES=normalized enrichment score.

Supplementary Figure 3

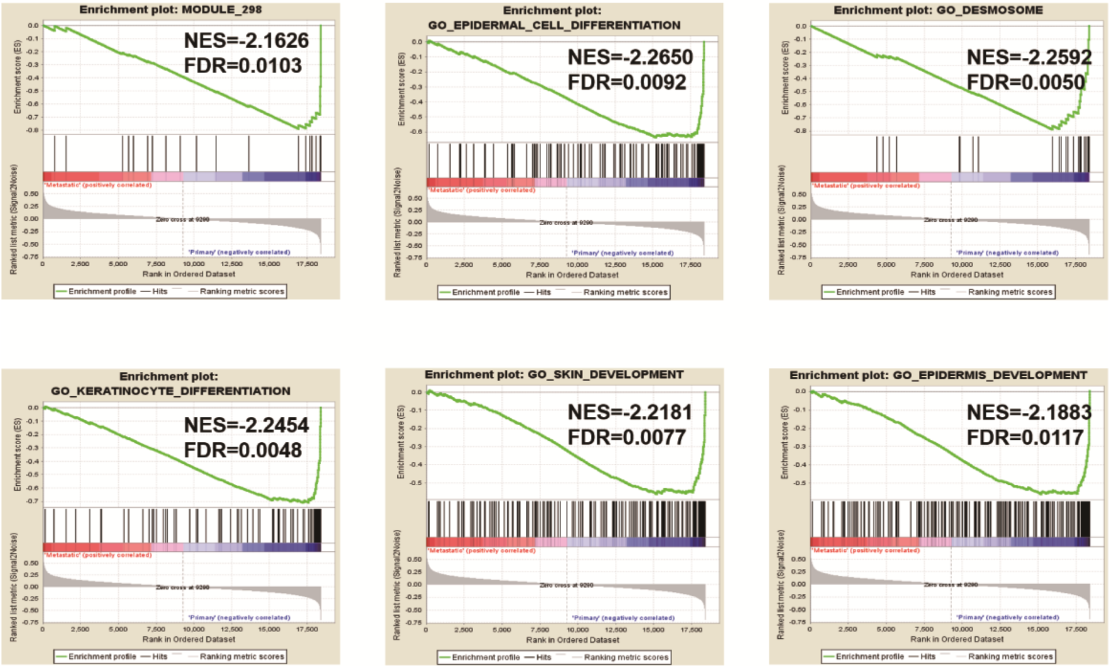

Gene set enrichment analysis (GSEA) for comparing genotype between metastatic and primary. FDR=false discovery rate; NES=normalized enrichment score.

Supplementary Figure 4

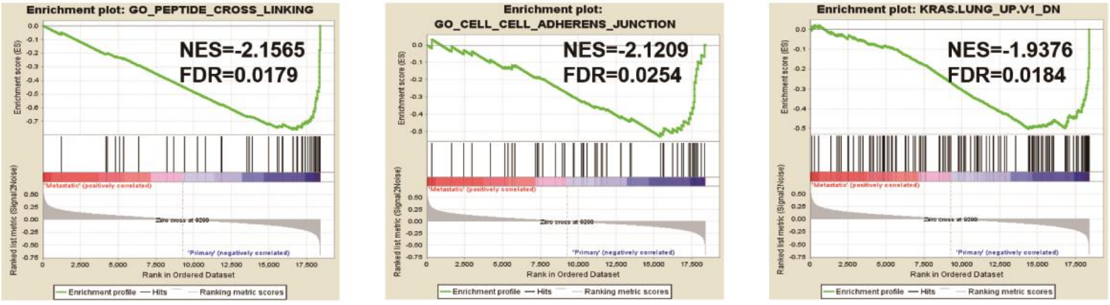

Gene set enrichment analysis (GSEA) for comparing genotype between metastatic and primary. FDR=false discovery rate; NES=normalized enrichment score.

Supplementary Figure 5

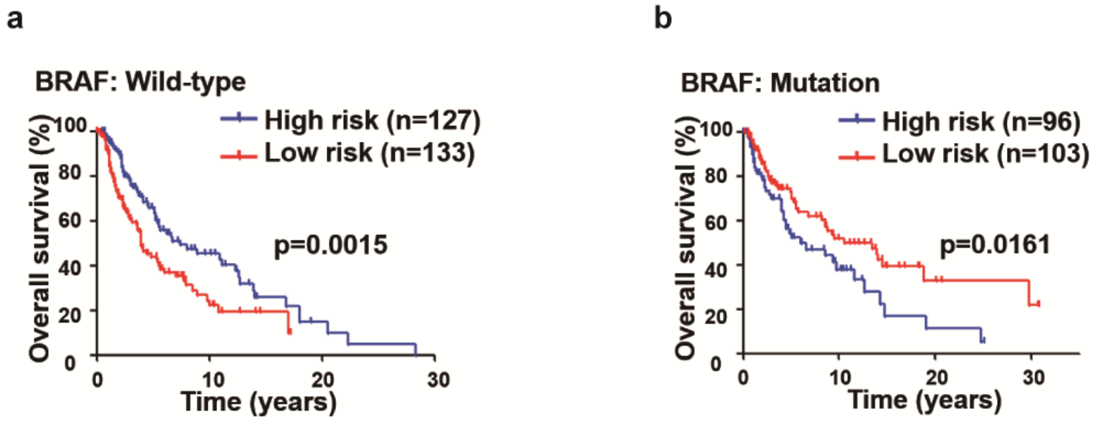

(a) Survival curves of overall survival for high and low risk groups classified by the local gene signature in BRAF wild-type melanoma patients (TCGA database). (b) Survival curves of overall survival for high and low risk groups classified by the local gene signature in BRAF mutation melanoma patients (TCGA database).
